# Reference Equations for the Six-Minute Walk Distance in the Healthy Chinese Han Population, Aged 18–30 Years

**DOI:** 10.1186/s12890-017-0461-z

**Published:** 2017-08-29

**Authors:** He Zou, Jia Zhang, Xiaoshu Chen, Yi Wang, Wei Lin, Jianfeng Lin, Hao Chen, Jingye Pan

**Affiliations:** 1 Department of Cardiovascular Medicine, Wenzhou People’s Hospital, the Wenzhou Third Clinical Institute Affiliated with Wenzhou Medical University, Wenzhou, Zhe Jiang Province China; 2 Department of Inspection Medical, Wenzhou People’s Hospital, the Wenzhou Third Clinical Institute Affiliated with Wenzhou Medical University, Wenzhou, Zhe Jiang Province China; 3 Department of General and Intensive Care Medical, The First Affiliated Hospital of Wenzhou Medical University, Wenzhou, Zhe Jiang Province China

**Keywords:** Six-minute Walk Test, Six-minute Walk Distance, Reference Equations, Healthy Subjects

## Abstract

**Background:**

The six-minute walk test (6MWT) is a safe, simple, inexpensive tool for evaluating the functional exercise capacity. However, there is a lack of standard reference equations for the six-minute walk distance (6MWD) in the healthy Chinese Han population with an age of 18–30 years. The aims of the present study were as follows: 1) to measure the anthropometric data and the walking distance in a sample of healthy Chinese Han population, aged 18–30 years; 2) to construct reference equations for the 6MWD; 3) to compare the measured 6MWD of our cohort with previously published equations.

**Methods:**

The anthropometric data, demographic, lung function and the walking distance of Chinese Han population, aged 18–30 years, were prospectively measured using a standardized protocol. Informed consent was obtained from each participant and the approval was obtained from the ethics committee of Wenzhou People’s Hospital. The 6MWT was performed twice and the longer 6MWD was used for further analysis.

**Results:**

A total of 355 subjects (176 female and 179 male) completed the 6MWT, and the average walking distance was 627.3 ± 52.88 m. The walking distance was achieved by females compared with males (607.4 ± 51.00 m vs. 646.9 ± 47.15 m; *p* < 0.0001) and active subjects compared with non-active subjects (646.1 ± 48.27 m vs. 611.6 ± 51.52 m; *p* < 0.0001). Univariate analysis showed age, height, body mass index, resting blood pressure, heart rate and blood pressure after the walk test and difference in heart rate before and after the walk test were significantly correlated with the 6MWD. Stepwise multiple regression analysis showed that height and difference in heart rate before and after the walk test were independent predictors associated with the 6MWD. The reference equations from Caucasian, Canadian and Chilean populations tend to overestimate the walking distance in our subjects, while Brazilian and Arabian equations tend to underestimate the walking distance. There was no significant difference in the walking distance between Korean equations and the current study.

**Conclusion:**

In summary, height and difference in heart rate before and after the walk test were the most significant predictors of the 6MWD, and the regression equations could explain approximately 38% and 31% of the distance variance in the female and male groups, respectively.

## Background

The 6MWT is a safe, simple, inexpensive tool for evaluating the functional exercise capacity of the individual to perform activities of daily living [1, 2]. The application of field walk tests in subjects with cardiopulmonary diseases results from the adaptability of the 12-min run fitness test developed by Cooper [3]. They developed the test to check the level of the USA Armed Forces personnel’s physical fitness. The test requires subjects to run the longest distance as soon as possible in 12 min in its original format. McGavin et al. [4] turned Cooper’s run fitness test into the 12-min walk test in the 70’s, the goal here was to assess the exercise tolerance of participants with chronic bronchitis. The 12-min walk test was changed to more simple (e.g. two-minute walk test and six-minute walk test), because the 12-min walk distance was difficult to complete for these participants with respiratory diseases [5]. But the responsiveness of the 2-min walk test tends to be limited, especially in less debilitated patients [6]. So in this case, the 6MWT became the most popular among these walk tests. In the past ten years, the 6MWT was generally used to evaluate the functional capacity, measure the effectiveness of several treatments and assess the prognosis of patients with cardiopulmonary diseases [7]. In recent years, the 6MWT has been used for clinical work in several other diseases, such as fibromyalgia, stroke, amputations, morbid obesity, Down syndrome, Alzheimer, cerebral palsy, etc. [8–14]. It is very important to develop reference equations for the 6MWD in healthy population. The 6MWT is a self-paced test. There are many factors that affect the 6MWD, such as different population, energy expenditure, operator encouragement, participant’s enthusiasm, etc. [7], so the American Thoracic Society (ATS) recommends researchers to establish reference values for each population [7]. There is a lack of standard reference equations for the 6MWD in the healthy Chinese Han population, aged 18–30 years.

The purpose of this study were as follows: 1) to measure the anthropometric data and the walking distance in a sample of healthy Chinese Han population, aged 18–30 years, 2) to construct reference equations for the 6MWD, 3) to compare the 6MWD of our cohort with previously published equations.

## Methods

### Subjects

We collected data over a 21-month period from November 2012 to July 2014, healthy subjects aged between 18 and 30 years were recruited from randomly selected local technical college and university. In front of the recruiting, each subject needed to a talk to understand the purpose of the study, as well as finish a questionnaire to exclude diseases to help ensure health. We provided formal consent for each subject and had obtained approval for the study from the ethics committee of Wenzhou People’s Hospital.

**The following were exclusion factors for our research entry:**

• presenting with any self-reported disease (cardiac disease, pulmonary disease, blood diseases, kidney disease, metabolic diseases, musculoskeletal disorders, cardiopulmonary diseases, neuromuscular diseases, etc).

• underweight or obese.

• basic heart rate ≥ 100 bpm or < 50bpm.﻿﻿

• basic systolic blood pressure ≥ 140 mmHg or diastolic blood pressure ≥ 90 mmHg.

• having respiratory symptoms or a common cold within the last one month.

• having cigarette smoking history.

• having any problem in walking or requiring the use of walking aids.

### Physical activity questionnaires

A questionnaire survey was conducted to understand the type, frequency, and duration of nearly a month exercise activity the subjects took part in the study. If the participant engaged in lower limb exercise for a minimum of 20 min per session, at least 3 times per week, the participant was classified as “active”. [15]. Those not meeting the above criteria were classified as “non-active”.

### Physical examination

The age, weight, height, BMI were measured before the test. The age was verified by the identity card. The height gauge was used for measuring the height without shoes while the participant’s back was straight. Weight (kg) was measured using an electronic scale and the BMI was calculated for each subject as BMI = weight/height^2^ (expressed in kg/m^2^). The following definitions were adopted [16]: extreme obesity (BMI ≥ 40 kg/m^2^); marked obesity (35 kg/m^2^ ≤ BMI ≤ 39.9 kg/m^2^); moderate obesity (30 kg/m^2^ < BMI < 34.9 kg/m^2^); overweight (25 kg/m^2^ ≤ BMI ≤ 29.9 kg/m^2^); normal weight (18 kg/m^2^ ≤ BMI ≤ 24.9 kg/m^2^) and underweight (BMI < 18 kg/m^2^).

### Pulmonary assessment

Lung function was measured using a standard portable spirometer (Vitalograph alpha, Ireland) that was calibrated on a daily basis. The Forced Expiratory Volume in one second (FEV_1_), forced vital capacity (FVC) and FEV_1_/FVC were measured before the test. Each participant was required to be done at least three measures; then, we recorded the largest value to analysis as recommended by the guidelines of the American Thoracic Society [17].

### Six-minute walk test

The 6MWT was conducted according to the standardized protocol [7]. The 6MWT was accomplished along a straight, long, flat, enclosed 30-m corridor with a hard surface that seldom went past. The operator put a mark each 3 m of the course. The turnaround points were marked with two orange traffic cones. The starting line indicated the beginning and end of each 60 m, which was marked by brightly coloured tape on the floor. To minimize the biological rhythms and temperature effects, the 6MWT was accomplished between 9:00 a.m. and 16:00 p.m. and the temperature rang was between 20 and 25 °C. The participants were required to avoid strenuous exercise and eat a light meal within 2 hours before the start of the test, and sat in a chair located near the starting line for at least 10 minutes before the start of the test. The oxygen saturation (SpO_2_), systolic and diastolic blood pressure and heart rate before the test were measured and the maximum heart rate (mHR= 220 - age) was calculated by the operator in ten minutes. Each subject was informed that the aim of the test that was to know how far they could walk at their own pace within the prescribed period of time. Then, each subject was asked to walk up and down the hallway as fast as possible in six minutes. If subjects had the symptoms of dizziness, leg cramps, chest pain or dyspnea, they could stop off half way to rest for a while, when they were well again, they were encouraged to continue walking as soon as possible. The 6MWT was monitored by a single operator, who recorded time and measured 6MWD by using of an electronic timer and a 30 m long meter scale. The operator needed to remind subjects every 60 s by using a standardized encouragement [7] (“you are doing well, you have five minutes left”, “good job, there are four minutes left”). The distance covered over the six minutes was recorded as the 6MWD. Each subject’s oxygen saturation, systolic and diastolic blood pressure, heart rate were measured at the end of the test by the operator. Each individual completed 6MWT twice and the second test was completed two hours later.

### Data analysis

The main variables in the research were on the normal distribution curve and were assessed by the Kolmogorov-Smirnov test such that the data were presented as means with standard deviations (SDs). We assessed the associations between 6MWD and the categorical variables (activity and gender) by the independent student’s *t* test. Unpaired student’s *t* test was used to compare between two tests. Repeatability of the 6MWD was examined using intraclass correlation coefficient (ICC). Potential factors, including age, weight, height, BMI, oxygen saturation systolic, heart rate, blood pressure and change in the oxygen saturation and heart rate before and after the test were assessed for the association with the 6MWD using first univariate analysis with the Spearman’s correlation test and then coordinated multivariate analysis by the forward stepwise multiple linear regression. The candidate variable was added to the model at each step and the process continued until no further significant contributing factor could be added. Its entry and removal from the model depended on whether the *P*-value was greater than 0.05, and the collinear between the multivariate was detected by variance inflation factors. We compared the individually measured 6MWDs with the distances predicted from six other countries published equations [18–23] for the same age ranges as in the corresponding research by the paired sample *t* test. Data analyses were performed by SPSS for Windows statistical software (version 15.0; SPSS, Inc., Chicago, IL). A *p*-value of <0.05 was considered significant in all analyses.

## Results

### Demographic, anthropometric and lung function

In the study, a total of 395 healthy subjects were recruited from November 2012 to July 2014. Forty subjects were excluded from the study, two subjects had foot sprain, one subject had a history of physician-diagnosed congenital heart disease, three subjects were presented with a resting systolic blood pressure ≥ 140 mmHg or resting diastolic blood pressure ≥ 90 mmHg, three subjects had obesity, five subjects had respiratory symptoms and twenty-six subjects had smoking history. Finally, 355 subjects (176 females and 179 males) completed the 6MWT, nobody prematurely terminated the test or needed to rest for a while during the test. Their characteristics of 355 subjects are summarized in Table 1. The mean age, height, weight and BMI of the cohort in the research were 23.9 ± 3.76 years, 166.6 ± 8.03 cm, 59.9 ± 10.35 kg and 21.5 ± 3.01 kg/m^2^, respectively. Females were significantly shorter and lighter than males and there was a sex difference in BMI. Less than half (45%) of the healthy subjects reported performing lower extremity exercise for at least 20 min each time, 3 times per week in the one month before the study. All the subjects had normal lung function. The values of FEV_1_ (forced expiratory volume in one second) were higher than 80%, and the values of FEV_1_/FVC (forced vital capacity) were higher than 70% of the normal predicted value. There were significant differences in the FVC (L), FEV_1_ (L), and FEV_1_ (% pred) between female and male subjects.

**Table 1:** Characteristics of the study subjects

Characteristic	Females (*n* = 176)	Males (*n* = 179)	*p*-value*	Total (*n* = 355)
Active	62	100		162
Age, years	23.8 ± 3.77	23.9 ± 3.76	NS	23.9 ± 3.76
Height, cm	160.4 ± 5.12	172.6 ± 5.4	<0.001	166.6 ± 8.03
Weight, kg	54.6 ± 8.78	65.0 ± 9.11	<0.001	59.9 ± 10.35
BMI, kg/m^2^	21.2 ± 3.19	21.9 ± 2.80	0.046	21.5 ± 3.01
FEV_1_, L	3.4 ± 0.38	4.5 ± 0.49	<0.001	3.9 ± 0.43
FVC, L	3.5 ± 0.40	4.8 ± 0.56	<0.001	4.2 ± 0.48
FEV_1_, % pred	97.1 ± 11.18	93.7 ± 10.76	0.024	95.4 ± 10.97

Values are expressed as mean ± SD

*BMI* Body mass index, *FVC* Forced vital capacity, *FEV*_1_ Forced expiratory volume in one second

*p-value between males and females

### Six-minute walk distance

**Fig. 1:**
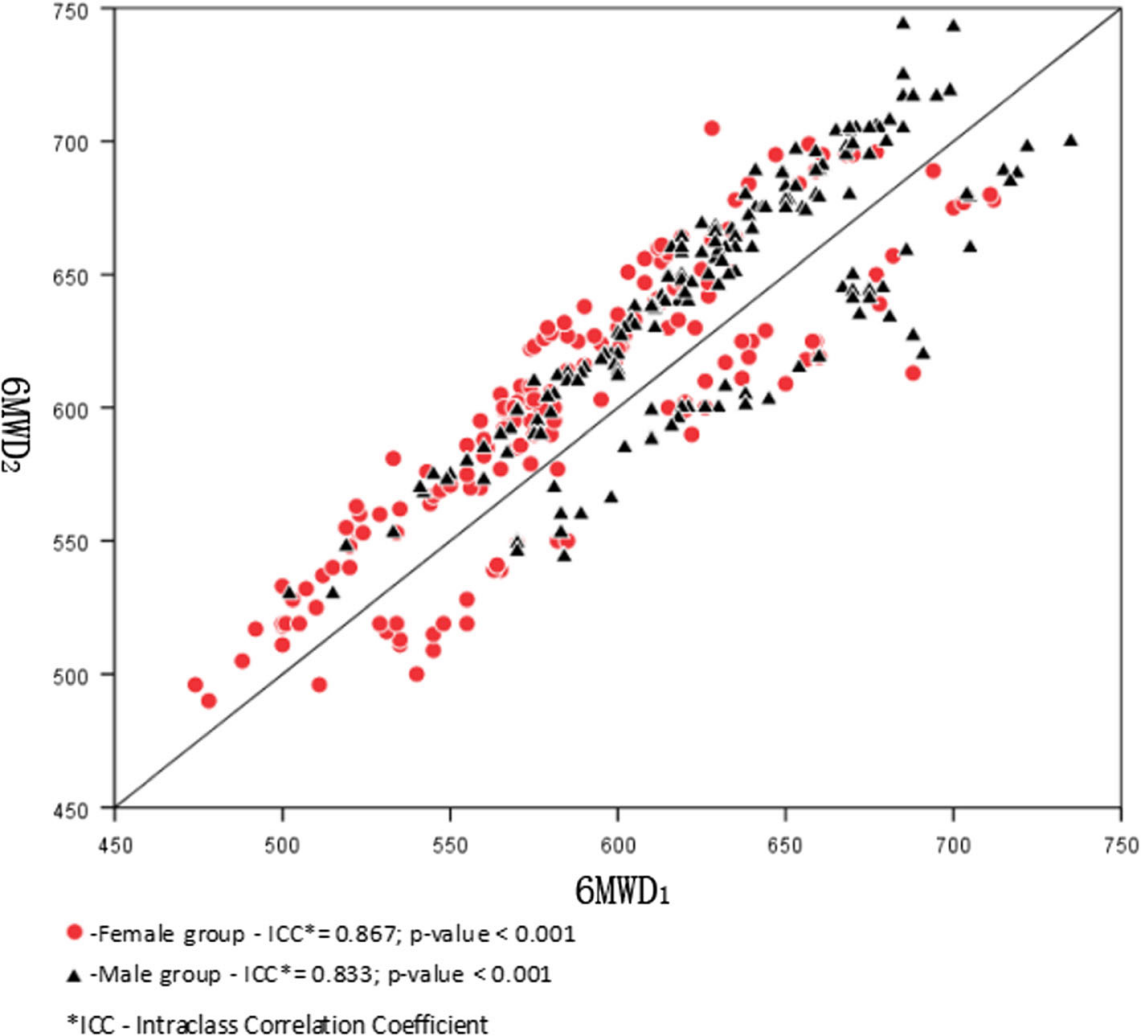
﻿Reproducibility of the walking distance between the first and second six-minute walk tests

The 6MWT data of 355 subjects are shown in Table 2. The mean 6MWD was 627.3 ± 52.88 m (range, 490.0–744.0 m) for the total group; it was 646.1 ± 48.27 m (range, 490.0–719.0 m) for the non-active group and 611.6 ± 51.52 m (range, 505.0–744.0 m) for the active group, with significantly shorter distances walked by the non-active group than by the active group (*p* < 0.001). The mean 6MWD was 607.4 ± 51.00 m (490.0–712.0 m) for the female group and 646.9 ± 47.15 m (530.0–744.0 m) for the male group. There were significantly shorter distances between the female and male groups (*p* < 0.05). The 6MWD increased significantly between test 1 and test 2 (607.5 ± 52.69 m vs. 620.4 ± 53.67 m, *p* < 0.05). Figure 1 demonstrates the reproducibility between two 6MWTs. The intraclass correlation coefficient between two 6MWTs for the female and male groups were very high (ICC = 0.867, ICC = 0.833, respectively) demonstrating good reproducibility.

**Table 2:** 6MWT results for the study subjects

Characteristic	Females (*n* = 176)	Males (*n* = 179)	*p*-value*	Total (*n* = 355)
Activity	624.9 ± 49.01	659.2 ± 43.02	<0.001	646.1 ± 48.27
Non-activity	597.9 ± 49.72	631.3 ± 47.79	<0.001	611.6 ± 51.52
Resting HR, bpm	76.2 ± 7.28	73.6 ± 8.38	0.002	74.9 ± 7.95
Resting SpO_2_, %	98.7 ± 0.97	97.9 ± 1.06	<0.001	98.4 ± 1.08
Resting SBP, mmHg	110.8 ± 8.51	114.2 ± 6.77	<0.001	112.5 ± 7.86
Resting DBP, mmHg	71.4 ± 8.27	72.0 ± 6.88	NS	71.7 ± 7.60
6MWD_1_, m	586.8 ± 50.85	627.8 ± 46.26	<0.001	607.5 ± 52.69
6MWD_2_, m	601.3 ± 51.63	639.2 ± 48.87	<0.001	620.4 ± 53.67
Best 6MWD, m	607.4 ± 51.00	646.9 ± 47.15	<0.001	627.3 ± 52.88
Borg after the 6MWT	0.23 ± 0.43	0.22 ± 0.44	NS	0.22 ± 0.44
HR after the 6MWT, bpm	118.7 ± 22.45	112.8 ± 24.06	0.017	115.7 ± 23.43
% mHR after the 6MWT	60.5 ± 11.13	57.5 ± 12.0	0.015	59.0 ± 11.64
Difference in HR, bpm	42.5 ± 21.52	39.2 ± 22.71	NS	40.8 ± 22.16
SpO_2_ after the 6MWT, %	98.3 ± 1.01	97.6 ± 1.13	<0.001	98.0 ± 1.10
Change in SpO_2_, %	−0.43 ± 0.66	−0.35 ± 0.73	NS	−0.38 ± 0.71
SBP after the 6MWT, mmHg	125.6 ± 10.22	133.3 ± 12.33	<0.001	129.5 ± 11.97
DBP after the 6MWT, mmHg	79.6 ± 7.34	81.7 ± 8.60	0.011	80.7 ± 8.06

Values are expressed as the mean ± SD

*6MWT* Six-minute walking test, *6MWD* Six-minute walking distance, *HR* Heart rate, *SpO*_2_ Oxygen saturation

*%mHR* Percentage of the predicted maximum heart rate, *SBP* Systolic blood pressure, *DBP* Diastolic blood pressure

*p-value between males and females

Female and male subjects reached 60.5% and 57.5% of their maximum predicted heart rates, respectively, at the end of the test. We found a significant difference between the female group and male group in terms of the resting systolic blood pressure, resting heart rate, resting oxygen saturation, oxygen saturation systolic after the test, systolic blood pressure and diastolic blood pressure after the test. We did not observe clinically significant differences in the resting diastolic blood pressure, Borg values, change in the oxygen saturation and heart rate before and after the test.

### Associations with the six-minute walk distance

**Fig. 2:**
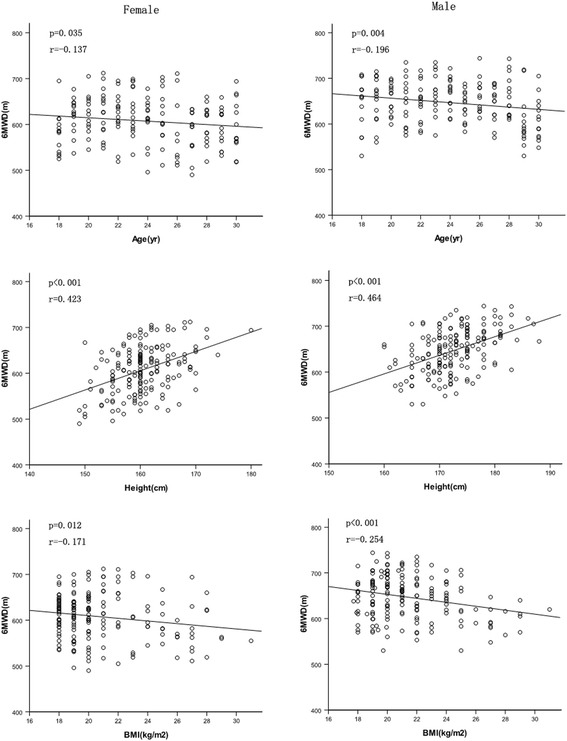
Relationship be﻿tween the 6MW﻿D and age, height and BMI for the female and male groups

**Fig. 3:**
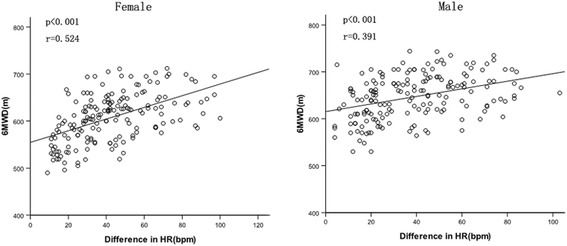
R﻿elationship between the 6MWD and difference in HR before and after the 6MWT for the female and male groups

By the Pearson correlation, the 6MWD significantly correlated with age, height, BMI, heart rate after the test, difference in heart rate before and after the test, resting systolic blood pressure, resting diastolic blood pressure, systolic blood pressure after the test, diastolic blood pressure after the test (Table 3). Figure 2 shows the relationship between the 6MWD and age, height and BMI in the female and male groups. The 6MWD-related variables such as age, height, difference in heart rate before and after the test and other related variables were used in the stepwise multiple regression analysis, it was found that difference in heart rate before and after the test and height were the most significant predictors of the distance (Fig. 3), which explained 38% and 31%, respectively, of the distance variance in the female and male groups (Table 4).

**Table 3:** Univariate correlation coefficients for the 6MWD

Variable	Females (*n* = 176)		Males (*n* = 179)	
	r-value	*p*-value	r-value	*p*-value
Age	−0.137	0.035	−0.196	0.004
Height	0.423	<0.001	0.464	<0.001
Weight	0.049	NS	−0.016	NS
BMI	−0.171	0.012	−0.254	<0.001
Resting HR	−0.049	NS	−0.063	NS
HR after the 6MWT	0.486	<0.001	0.351	<0.001
Difference in HR	0.524	<0.001	0.391	<0.001
Resting SpO_2_	−0.077	NS	−0.012	NS
SpO_2_ after the 6MWT	−0.052	NS	0.047	NS
Change in SpO_2_	0.035	NS	0.093	NS
Resting DBP	−0.134	0.038	−0.165	0.014
Resting DBP	−0.111	0.071	−0.130	0.041
SBP after the 6MWT	0.271	<0.001	0.236	<0.001
DBP after the 6MWT	0.135	0.037	0.153	0.020

*6MWT* Six-minute walking test, *6MWD* Six-minute walking distance, *HR* Heart beat, *BMI* Body mass index

*SBP* Systolic blood pressure, *DBP* Diastolic blood pressure, *SpO*_2_ Oxygen saturation

r-value: Pearson’s correlation coefficient

**Table 4:** Stepwise multiple linear regression analysis by sex for factors associated with the 6MWD

	Female			Male		
	Unstandardized Coefficient	SE	*p*-value	Unstandardized Coefficient	SE	*p*-value
Constant	−0.458	94.924		−11.394	93.543	
Height, cm	3.494	0.595	<0.001	3.659	0.545	<0.001
Difference in HR	1.113	0.142	<0.001	0.692	0.129	<0.001
R square	0.394			0.326		
Change in R square	0.387			0.318		

Both height and the difference in heart rate before and after the walk test were significantly associated with 6MWD

The reference equations of the 6MWD are as follows:

Female: 6MWD (m) = − 0.458 + (difference in heart rate × 1.113) + [height (cm) × 3.494]; r^2^ = 0.387

Male: 6MWD (m) = −11.394 + (difference in heart rate × 0.692) + [height (cm) × 3.659]; r^2^ = 0.318

The reference equations of the 6MWD are as follows:

Female: 6MWD (m) = − 0.458 + (difference in heart rate × 1.113) + [height (cm) × 3.494]; r^2^ = 0.387.

Male: 6MWD (m) = − 11.394 + (difference in heart rate × 0.692) + [height (cm) × 3.659]; r^2^ = 0.318.

### Comparison with published regression equations

Comparisons between the measured 6MWD values in our subjects and predicted 6MWD values for the same age ranges from the reference equations derived in Brazilian [18], Arabian [19], Caucasian [20], Canadian [21], Chilean [22] and Korean [23] populations are shown in Table 5. The reference equations from Caucasian, Canadian and Chilean populations tend to overestimate the walking distance of our subjects, Chetta et al. [20] by 9.9 ± 54.64 m (*p* < 0.05), Gibbons et al. [21] by 129.1 ± 73.86 m (*p* < 0.05), Osses et al. [22] by 107.1 ± 46.89 m (*p* < 0.05), on the contrary, Brazilian and Arabian equations tend to underestimate it, Iwama et al. [18] by 17.9 ± 49.58 m (*p* < 0.05) and Alameri et al. [19] by 168.9 ± 45.29 m (*p* < 0.05). There was no significant difference in the walking distance between Korean equations and the current study, Kim et al. [23] by 4.9 ± 55.40 m (*p* > 0.05).

**Table 5:** Measured 6MWD and predicted 6MWD for the same age range based on the equations reported in previous studies

Study	Measured (m)	Predicted (m)	Measured − predicted (m)
Iwama et al. [18]	627.3 ± 52.88	609.4 ± 31.53	17.9 ± 49.58*
Alameri et al. [19]	627.3 ± 52.88	458.3 ± 22.86	168.9 ± 45.29*
Chetta et al. [20]	627.5 ± 52.65	637.4 ± 16.41	−9.9 ± 54.64*
Gibbons et al. [21]	627.5 ± 52.65	756.7 ± 38.70	−129.1 ± 73.86*
Osses et al. [22]	627.5 ± 52.65	737.4 ± 34.88	−107.1 ± 46.89*
Kim et al. [23]	625.1 ± 53.60	620.1 ± 15.8	4.9 ± 55.40

*6MWD* Six-minute walking distance

*p < 0.05 according to Student's t-test

## Discussion

To our knowledge, this is the first study to predict the 6MWD in the healthy Chinese Han population, aged 18–30 years.

**Fig. 4:**
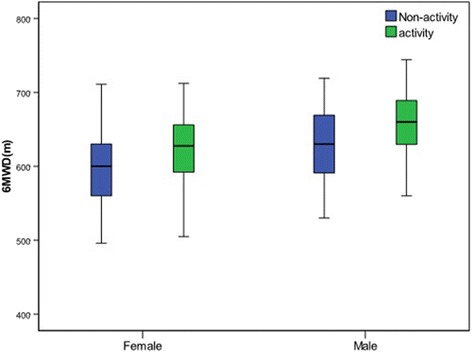
﻿Relationship between the 6MWD and activity for the female and male groups﻿

There was a significant difference in the distance walked between the female group and male groups in our study (Fig. 4). The male group walked a greater average distance walked than the female group, which was possibly because they are taller and have higher levels of physical activity and a greater muscle mass.

There was a significant difference in the distance walked between the activity group and non-activity groups in our study (Fig. 4). Studies of exercise physiology showed that physical exercise had a significant positive correlation with muscle strength [24]. Conversely, a sedentary lifestyle usually alters the muscle metabolism, muscle mass and physical capacity [24], which could explain why the average distance walked in the activity group was significantly higher than that of the non-activity group in our study.

A modest “learning effect” was apparent in the 355 healthy subjects between the first and second 6MWTs. The learning effect may be due to improved coordination, overcoming anxiety and finding optimal stride length to influence walking distance [25].

We found that age had negative correlation with the 6MWD. This could be a reason that their muscle mass, muscle strength and maximal oxygen uptake gradually decreased with age. We observed that height was strongly correlated with the distance walked and was the predominant variable in the regression equation for subjects. This may be because the taller a person is, the longer the strides, which makes walking more efficient and probably results in a longer distance walked. In contrast, our researchers found a negative correlation between BMI and the distance walked in our study, but BMI was not represented in the final regression equation. Our researchers also found that weight was not associated with the distance walked in our study. The possible reason may be that the subjects in our study were recruited from local technical college and university, and their weights were within the normal range. We observed that difference in heart rate before and after the walk test was significantly positively correlated with the distance walked in our study, and was represented in the final regression equation. This may be because difference in heart rate before and after the test represents the level of effort expended by a subject to perform the test.

There were also significant correlations between the 6MWD and resting blood pressure and blood pressure after the test, but these were not represented in the final regression equation. The reason was that our subjects were healthy population, and were free of high blood pressure and heart disease.

The mean 6MWD in our study was 627.3 ± 52.88 m. The forward stepwise multiple linear regression for the female and male groups showed that subjects’ difference in heart rate before and after the test and height were the most significant predictors of the 6MWD, and the regression equations could explain approximately 38% and 31% of the distance variance, respectively.

The previous published studies [18–23] did not reliably predict 6MWD in our population. The reference equations were Caucasian [20], Canadian [21] and Chilean [22] populations tended to overestimate the walking distance in our subjects. The Korean [23] equation was estimated similarly to our population, while the reference equations were from Brazilian [18] and Arabian [19] equations tended to underestimate the distance. These differences are commonly caused by differences in the anthropometric factors, demographic characteristic, racial background of the recruited subjects and the standardization was executed in each study. In addition, participants’ level of daily physical activity and attitude toward the study should also be as a kind of possibility. Our study complied with the American Thoracic Society guidelines [7]. For example, the 6MWT was performed twice by each participant and the longer 6MWD was used for further analysis in our study. There were some demands on the technical aspects according to the guidelines to ensure the execution of the 6MWT, particularly in the 30 m length of the corridor and one practice walk. However, some studies did not consider the differences in these methodological aspects of the 6MWT. For example, in the study by Gibbons et al. [21] a 20-m corridor was used, while in the study by Alameri et al. [19] a practice test was not performed. In four studies [18, 20, 22, 23] consistent with our protocol. Our subjects reached an average of 59 ± 11% of their maximum predicted heart rates (mHRs), these subjects in the study by Chetta et al. [20] and Osses et al. [22] reached respectively an average of 67 ± 10% and 74% of their mHRs, so their average 6MWD were significantly longer than those of our study. Conversely, the male and female subjects in the study by Alameri et al. [19] reached respectively an average of 44% and 47% of their mHRs, so the average 6MWD was significantly shorter than that of our study. Though these subjects in the study by Iwama et al. [18] reached 65 ± 13% of their mHRs, these subjects are heavier than those in our study. In addition to the subjects’ levels of daily physical activity, their attitude and psychological factors may also influence the degree of effort [26].

But, our research has also certain insufficiency. First, our subjects consisted of young people, we did not recruit individuals aged who were older than 30 years of age. The 6MWT has clinical utility in several diseases such as cardio-respiratory dysfunction’s disease, which occurs in the elder population, and our reference equations are not available for these populations. However, equally important are clinical practice that provide reference equations for the six-minute walk distance in different age groups, because young people’s reference equations for the six-minute walk distance are different from that of middle-aged and older people and some diseases also occur in healthy young people. Second, in the current study, the sample is college students, teachers and workers from local technicalcollege and university and may not be representative of the entire Chinese population. Third, an important limitation is the role of heart rate in the reference equations. Those participants in whom an elevated heart rate was detected after the 6MWT may actually represent less-fit participants. A large multi-center study is needed to solve these problems.

## Conclusions

In summary, the height and difference in heart rate before and after the walk test were the most significant predictors of the 6MWD, and the regression equations could explain approximately 38% and 31% of the distance variance by the female and male groups, respectively.

## Abbreviations

6MWD: Six-minute walk distance
6MWT: Six-minute walk test
ATS: The American Thoracic Society
BMI: Body mass index
BP: Blood pressure
DBP: Diastolic blood pressure
FEV_1_: Forced expiratory volume in one second
FVC: Forced vital capacity
HR: Heart rate
mHR: The maximum heart rate
SBP: Systolic blood pressure
SD: Standard deviation
SpO_2_: Peripheral oxygen saturation
